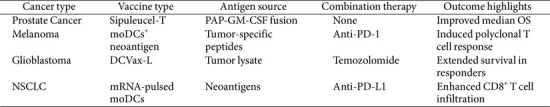# Refining Dendritic Cell-Based Cancer Vaccines: Subset Targeting, Translational Barriers, and Emerging Strategies

**DOI:** 10.4014/jmb.2506.06021

**Published:** 2025-08-12

**Authors:** Inmoo Rhee

**Affiliations:** Department of Bioscience and Biotechnology, Sejong University, Seoul 05006, Republic of Korea

**Keywords:** Dendritic cells (DCs), DC vaccine, cancer immunotherapy

## Abstract

Dendritic cells (DCs) are pivotal regulators of immune responses, capable of initiating robust adaptive immunity through antigen presentation. As the most potent antigen-presenting cells, they have emerged as central components of cancer immunotherapy. Over the last decade, advances in molecular engineering, bioinformatics, and nanomedicine have transformed the design of DC-based vaccines. Strategies now include personalized neoantigen loading, mRNA-electroporation, nanoparticle-mediated delivery, and combinatorial regimens with immune checkpoint inhibitors. In addition, emerging approaches that target DC subsets *in vivo*, especially cDC1s, have demonstrated enhanced efficacy in preclinical and early clinical studies. This review provides a comprehensive overview of the biological roles of DCs and evaluates the evolution of DC vaccine platforms while also highlighting new technologies and clinical insights that aim to tumor-induced immunosuppression suppression and improve therapeutic outcomes.

## Introduction

Dendritic cells function as professional antigen-presenting cells and serve as essential coordinators between the innate and adaptive immune systems. This immunological role of DCs was first identified by Steinman and Cohn in 1973 [1]. DCs reside in peripheral and lymphoid tissues and are equipped to internalize antigens, undergo maturation, and activate naive T cells. DCs can precisely recognize exogenous antigens and present them to T cells, inducing antigen-specific immune responses. This makes them well suited as vaccine platforms for use in educating the immune system against pathogens or tumors.

**Fig. 1:**
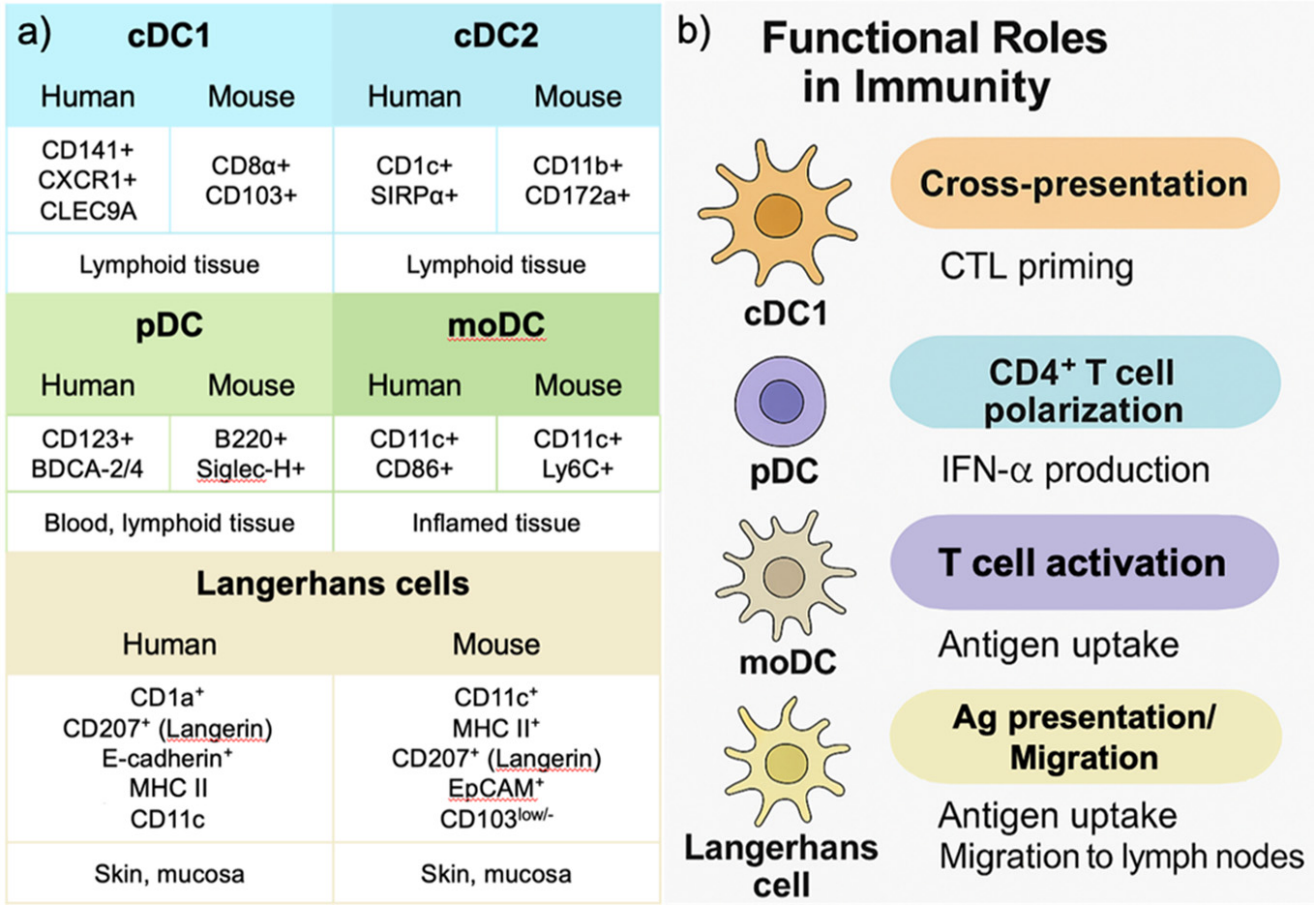
Functional subsets and functional roles of dendritic cells in humans and mice. (**A**) Summary of major dendritic cell (DC) subsets in humans and mice. The table lists conventional type 1 DCs (cDC1s), conventional type 2 DCs (cDC2s), plasmacytoid DCs (pDCs), monocyte-derived DCs (moDCs), and Langerhans cells, with their key markers and typical tissue distributions. (**B**) Functional specialization of each DC subset. cDC1s mediate cross-presentation and cytotoxic T lymphocyte (CTL) priming; pDCs produce IFN-α; moDCs efficiently uptake antigens and activate T cells; Langerhans cells participate in antigen uptake and migration to lymph nodes.

Recent studies have described significant heterogeneity among DC subsets, including conventional type 1 DCs (cDC1s), conventional type 2 DCs (cDC2s), plasmacytoid DCs (pDCs), and monocyte-derived DCs (moDCs)(Fig. 1A, Table 1). Among these, cDC1s show exceptional ability to cross-present tumor antigens and activate cytotoxic T lymphocytes, making them ideal targets for vaccine strategies [2]. In particular, DCs can present tumor-derived antigens to activate CD8^+^ T cells, which enable direct cytotoxic responses against cancer cells and highlight their therapeutic potential in cancer immunotherapy. Traditional DC vaccines used moDCs pulsed with tumor lysates or peptides. These platforms demonstrated feasibility and safety but offered limited clinical success. The development of personalized DC vaccines based on tumor-specific neoantigens has addressed this issue. Advances in sequencing and epitope prediction allow precise identification of immunogenic mutations, which can be introduced into DCs using synthetic peptides or mRNA constructs [3-5].

Electroporated mRNA improves antigen expression and promotes DC maturation [4]. Nanoparticle-based delivery vehicles enhance lymph node trafficking and co-delivery of tumor antigens with immune stimulants [6]. These approaches have been successfully combined with immune checkpoint blockade, particularly anti-PD-1 and anti-CTLA-4 therapies, to enhance T cell activation and overcome immune tolerance.

However, several challenges persist. Tumor-derived factors such as VEGF, IL-10, and IDO can suppress DC function. Researchers continue to investigate ways to improve DC migration and survival, and to design vaccines that maintain potency in immunosuppressive environments. This review highlights key innovations in DC-based cancer vaccines and outlines clinical strategies that aim to optimize antitumor immunity.

## Biological Features of DC Subsets and Implications for Vaccine Design

### Migration and Tissue Positioning

The migratory behavior of DCs is essential for the initiation of immune responses. In their immature state, DCs patrol peripheral tissues where they efficiently capture antigens through macropinocytosis, receptor-mediated endocytosis, and phagocytosis. Immature DCs express high levels of antigen-capturing receptors, such as C-type lectins (*e.g.*, DEC-205, DC-SIGN) and pattern recognition receptors (PRRs), including Toll-like receptors (TLRs), which detect danger-associated molecular patterns [2, 7].

Following antigen uptake, DCs undergo a maturation process characterized by the upregulation of major histocompatibility complex (MHC) molecules and costimulatory markers (CD80, CD86, CD40). These changes equip DCs for the activation of T cells [8]. A crucial step in this transition involves the expression of the chemokine receptor CCR7, which directs mature DCs to draining lymph nodes via afferent lymphatics [9]. Once localized in the lymphoid tissues, mature DCs present processed antigens to naive T cells and initiate adaptive immune responses.

Studies have shown that DC migration and function are modulated by tissue microenvironments, stromal cell-derived signals, and inflammatory stimuli. For example, tissue-specific imprinting in mucosal sites involves local cytokines and integrin profiles [10]. Additionally, tumor-derived suppressive factors such as prostaglandin E2 (PGE2) and vascular endothelial growth factor (VEGF) impair DC migration and maturation, which diminishes their immunostimulatory potential [11, 12].

### Conventional/Classical DCs

Conventional or classical DCs (cDCs) represent a major subset of DCs that specialize in antigen presentation and T cell activation [13]. cDC1s differentiate from common DC progenitors under the control of transcription factors BATF3 and IRF8, which promote their lineage commitment and cross-presentation capacity. In contrast, cDC2 differentiation is driven by IRF4, which guides their development toward efficient CD4^+^ T cell priming and polarization. These subsets acquire additional functional characteristics based on their tissue-specific microenvironments. For instance, within the tumor microenvironment, cDC1s often become functionally suppressed due to inhibitory cytokines, such as IL-10 or TGF-β, resulting in reduced antigen presentation and impaired CD8^+^ T cell activation. These cells express high levels of CD11c and MHC class II and exhibit a dendritic morphology. In humans, two primary subsets of cDCs are identified: CD1c^+^ (BDCA-1) and CD141^+^ (BDCA-3) DCs (Table 1). CD1c^+^ DCs can prime CD4^+^ T helper cells and support Th1 or Th2 polarization based on contextual cues. In contrast, CD141^+^ DCs are efficient at cross-presenting antigens to CD8^+^ T cells and express CLEC9A and XCR1, which makes them suitable for targeted vaccine delivery [14, 15]. In murine models, cDCs are classified by the expression of CD8α or CD11b. CD8α^+^ cDCs predominantly localize to lymphoid organs and possess strong cross-presentation abilities similar to human CD141^+^ DCs. CD11b^+^ cDCs are enriched in peripheral tissues and promote MHC class II-mediated CD4^+^ T cell responses (Fig. 1B) [15]. Migratory cDCs, including CD103^+^ subsets in tissues such as the skin and intestine, play essential roles in peripheral immune surveillance and tolerance [16].

Targeting antigens specifically to cDC1 subsets-CD141^+^ in humans or CD8α^+^ in mice-improves vaccine efficacy by enhancing CD8^+^ T cell priming, especially when combined with checkpoint inhibitors or Toll-like receptor agonists [6].

### Plasmacytoid DCs (pDCs)

pDCs represent a specialized DC subset known for their ability to produce large quantities of type I interferons (IFNs) in response to viral nucleic acids. pDCs exhibit a plasma cell-like morphology and express surface markers, such as CD123, BDCA-2 (CD303), and BDCA-4 (CD304) in humans. In mice, they are identified by B220, Siglec-H, and BST2 [17]. Unlike cDCs, pDCs recognize viral RNA and DNA through endosomal Toll-like receptors TLR7 and TLR9. Upon stimulation, they secrete IFN-α, which contributes to antiviral defense, immune cell recruitment, and T cell activation [18]. This response enhances innate immunity and helps bridge the transition to adaptive immune activation.

Recent research has shown that tumors can subvert pDC function. In many cancers, tumor-derived signals inhibit IFN-α secretion, drive pDCs toward a regulatory phenotype, and promote the expansion of immunosuppressive regulatory T cells. This shift reduces effective antitumor immunity and correlates with poor clinical prognosis [19].

To restore their function, several strategies have been developed. TLR agonists such as CpG oligodeoxynucleotides can re-activate pDCs and stimulate antitumor responses. In addition, pDCs have demonstrated an ability to process and present antigens under optimized conditions. These features support their use as carriers for tumor vaccines in settings that require systemic IFN-mediated immune enhancement.

### Monocyte-Derived DCs (moDCs)

moDCs are a widely used subset in both research and clinical applications due to their accessibility and ease of generation. These cells differentiate from peripheral blood monocytes in the presence of GM-CSF and IL-4 and are typically generated *ex vivo*. moDCs express high levels of CD11c, MHC class II, CD80, CD86, and CD40 and exhibit potent antigen uptake and T cell stimulatory capacity [8].

In cancer immunotherapy, moDCs have served as the principal cell type for autologous DC vaccines [20]. Clinical protocols often involve the collection of patient-derived monocytes, their differentiation into moDCs, and subsequent loading with tumor antigens in the form of peptides, tumor lysates, or RNA. These antigen-loaded DCs are then reinfused to stimulate tumor-specific T cell responses. moDC-based vaccines have been tested in melanoma, prostate cancer, glioblastoma, and renal cell carcinoma, demonstrating safety and some degree of clinical benefit [21].

While autologous moDC vaccines remain the clinical standard due to their safety, immunological compatibility, and customizability, allogeneic moDC approaches have been explored as scalable alternatives [22]. Allogeneic DC vaccines can be produced as off-the-shelf products with standardized potency, but they carry a risk of reduced immunogenic specificity and potential alloreactivity, which limits their clinical application compared with autologous platforms.

Despite their advantages, moDCs have limitations. Compared to naturally circulating DC subsets such as cDC1s, moDCs display lower migratory capacity, reduced lymph node homing, and more heterogeneous gene expression profiles [23]. These characteristics may impair their ability to efficiently prime T cells *in vivo*.

To enhance their function, recent strategies have incorporated mRNA electroporation to induce expression of immunostimulatory cytokines (*e.g.*, IL-12p70) or chemokine receptors (*e.g.*, CCR7). Co-delivery of TLR ligands and nanoparticle-formulated adjuvants has also improved the immunogenicity of moDC vaccines [24]. Although the field has shifted focus toward physiologically specialized DC subsets, moDCs continue to provide a flexible platform for experimental optimization and personalized vaccine development.

### *Ex Vivo* Generation of DC Vaccines

**Fig. 2:**
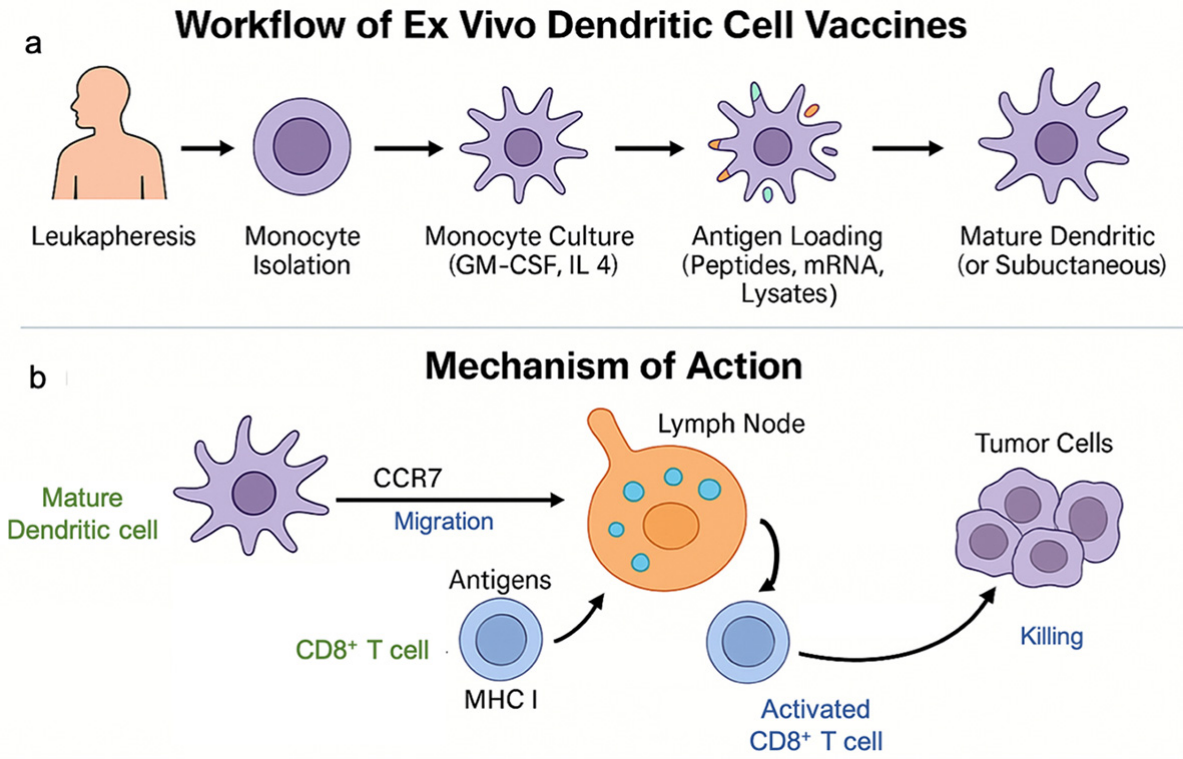
Workflow and mechanism of action of *ex vivo* DC vaccines. (**A**) The *ex vivo* generation of DC vaccines begins with leukapheresis to isolate peripheral blood mononuclear cells (PBMCs), followed by monocyte isolation and culture with granulocyte-macrophage colony-stimulating factor (GM-CSF) and interleukin-4 (IL-4) to induce differentiation into immature DCs. These cells are then loaded with tumor-associated antigens such as peptides, mRNA, or tumor lysates and matured using cytokines or Toll-like receptor ligands before administration. (**B**) Mature DCs migrate to draining lymph nodes through CCR7-mediated homing, present antigenic peptides on MHC class I molecules to CD8^+^ T cells, and induce their activation and clonal expansion. Activated cytotoxic T lymphocytes infiltrate tumors and mediate targeted tumor cell killing.

The *ex vivo* generation of dendritic cell (DC) vaccines begins with isolating peripheral blood mononuclear cells (PBMCs) from autologous leukapheresis (Fig. 2A). Monocytes are cultured with granulocyte-macrophage colony-stimulating factor (GM-CSF) and interleukin-4 (IL-4) to induce differentiation into immature DCs. These cells are then loaded with tumor-associated antigens, which can include defined peptides, tumor lysates, or messenger RNA encoding neoantigens [25]. Maturation is essential to enhance the antigen presentation capacity of DCs and to activate T cells effectively. This is achieved by exposing DCs to proinflammatory cytokines, such as TNF-α, IL-1β, and IL-6, or by stimulation with Toll-like receptor ligands, such as poly I:C or CpG, or CD40L. Mature DCs express high levels of costimulatory molecules (CD80, CD86, CD40), MHC class I and II molecules, and CCR7, enabling their migration to lymph nodes to initiate immune responses (Fig. 2B).

Recent protocols have incorporated mRNA electroporation to induce expression of tumor antigens or immunostimulatory molecules such as IL-12 or CD40L, enhancing DC-mediated T cell activation. Additional approaches have used co-electroporation of CCR7 to improve lymph node homing or small molecule inhibitors that block immunosuppressive pathways, such as IDO and TGF-β signaling, to increase vaccine potency [26]. While this standardized workflow under good manufacturing practice (GMP) conditions has supported the widespread clinical application of monocyte-derived DC (moDC) vaccines in cancer trials, *ex vivo*-generated moDCs often show reduced migratory capacity, altered cytokine secretion profiles, and less-efficient T cell priming compared with naturally circulating DC subsets. These limitations have driven research into next-generation vaccines that utilize specialized DC subsets or *in vivo* targeting strategies to achieve greater therapeutic efficacy.

## DC-Based Vaccines for Cancer Immunotherapy

### Evolution of DC Vaccine Platforms

DC-based vaccines have evolved significantly from early autologous approaches to more advanced and defined platforms. Initial clinical studies focused on moDCs that were generated *ex vivo* using GM-CSF and IL-4 and loaded with tumor-associated antigens (TAAs). Although moDC vaccines showed feasibility and safety, their clinical efficacy remained modest due to functional heterogeneity and limited lymph node migration [23].

Recent strategies have prioritized the use of more functionally specialized subsets, particularly conventional type 1 DCs (cDC1s), which possess a high capacity for antigen cross-presentation and IL-12 production [27]. These cells express key markers such as XCR1 and CLEC9A and induce robust CD8^+^ T cell responses. Clinical programs now explore the isolation or expansion of natural cDC1s for therapeutic use [28, 29].

Additionally, researchers have developed DCs from CD34^+^ hematopoietic stem cells or induced pluripotent stem cells (iPSCs), which provide scalable and reproducible vaccine platforms [30]. Standardized good manufacturing practice (GMP)-compliant protocols have facilitated clinical translation of these next-generation DC vaccines.

### Antigen Delivery Strategies

The method of antigen delivery significantly impacts DC vaccine efficacy. Early approaches employed whole tumor lysates or defined peptide pools, but these often failed to generate robust immune responses due to MHC restriction or poor immunogenicity. In contrast, personalized neoantigens identified through genomic sequencing are highly specific and exhibit low central tolerance, which enhances T cell recognition [31].

These neoantigens can be delivered using mRNA electroporation, which offers rapid, transient expression without genomic integration. This method allows co-delivery of multiple antigens and immunostimulatory molecules, thereby enhancing DC activation and antigen presentation [32]. Several clinical trials in melanoma and glioblastoma have shown that mRNA-pulsed DC vaccines elicit polyclonal T cell responses and correlate with improved progression-free survival [33].

Researchers have optimized antigen routing by linking tumor antigens to sequences that direct them to the MHC class I or II presentation pathways. DCs electroporated with mRNA encoding tumor antigens fused to lysosomal targeting domains, such as LAMP-1 or ubiquitination tags, exhibit enhanced immunogenicity. Co-electroporation with IL-12 or CD40L further promotes T cell polarization and cytotoxicity [34].

### *In Vivo* DC Targeting Strategies

*In vivo* DC targeting strategies aim to eliminate the need for *ex vivo* manipulation by delivering antigens directly to specific DC subsets within the body. This approach uses antibody-antigen conjugates or nanoparticles functionalized with ligands that recognize DC-specific receptors, such as DEC-205 (CD205), XCR1, or CLEC9A [35]. These receptors are highly expressed on cross-presenting cDC1s, which makes them ideal candidates for vaccine delivery.

Antigen delivery through anti-CLEC9A antibodies or chemokine fusions to XCL1 has successfully enhanced antigen uptake, cross-presentation, and CD8^+^ T cell activation. Adjuvants such as poly I:C or CpG oligodeoxynucleotides are often co-administered to trigger DC maturation and ensure effective antigen processing. These targeting systems increase the efficiency of T cell priming while minimizing off-target effects [36].

Nanoparticle-based carriers have further improved antigen delivery. These systems use liposomes, polymeric nanoparticles, or micelles to encapsulate tumor antigens and adjuvants. Surface modifications enhance lymphatic drainage and DC receptor targeting, and biodegradable cores provide controlled antigen release [16]. The addition of pH-sensitive elements promotes endosomal escape and efficient MHC class I antigen presentation.

RNA-lipoplex vaccines are another promising platform [37]. These carriers transport mRNA encoding tumor antigens systemically and preferentially accumulate in splenic DCs. RNA sensing through intracellular pattern recognition receptors initiates innate immune activation and enhances antigen presentation without additional adjuvants.

### Combination Therapies for Enhanced Immunity

DC vaccines have demonstrated modest clinical efficacy when used alone, especially in patients with established immunosuppressive tumor microenvironments. To address this limitation, researchers have combined DC vaccination with immune checkpoint inhibitors (ICIs) to reinvigorate exhausted T cells and improve response durability. Several trials in melanoma, glioblastoma, and hepatocellular carcinoma have reported increased tumor regression and survival following DC vaccine administration in conjunction with anti-PD-1 or anti-CTLA-4 antibodies [18, 19].

Radiotherapy and oncolytic viruses have also enhanced DC vaccine efficacy. Radiation promotes immunogenic cell death and releases tumor-associated antigens, which can be captured by endogenous or vaccine-delivered DCs. Oncolytic viruses such as HSV-1 and reovirus generate in situ danger signals and inflammatory cytokines that promote DC maturation and T cell recruitment.

The use of innate immune stimulators, such as STING agonists or TLR ligands, has provided additional tools to improve DC function in immunosuppressive environments. These agents trigger type I IFN production, which enhances antigen cross-presentation and CD8^+^ T cell priming. Researchers have also tested low-dose chemotherapy and regulatory T cell (Treg) depletion as methods to enhance vaccine responsiveness [20, 21].

Overall, rational combinations that align DC activation with T cell rescue and tumor sensitization offer the most promising clinical outcomes.

### Overcoming Tumor Microenvironment Barriers

The immunosuppressive tumor microenvironment (TME) poses a major challenge to effective DC-based immunotherapy. Tumors produce factors such as transforming growth factor-beta (TGF-β), interleukin-10 (IL-10), vascular endothelial growth factor (VEGF), and prostaglandin E2 (PGE2) that inhibit DC maturation and antigen presentation [11]. In addition, indoleamine 2,3-dioxygenase (IDO) expression and regulatory T cell expansion further suppress DC-driven T cell priming.

To counteract these effects, the expression of IL-12p70 or dominant-negative receptors for TGF-β in DCs enhances their capacity to activate cytotoxic T lymphocytes and resist tumor-mediated immunosuppression [38]. Some protocols incorporate small interfering RNA (siRNA) targeting IDO or co-electroporation with activating molecules, such as CD40L or constitutively active IKKβ. These modifications restore DC immunogenicity and support T cell polarization *in vivo*.

Innate immune pathway activation through STING or TLR agonists provides another strategy to reprogram tolerogenic DCs. STING agonists activate type I IFN pathways that promote cross-priming and reinforce antitumor immunity [39]. Similarly, poly I:C and CpG ODNs enhance antigen processing and costimulatory molecule expression. These adjuvants can be co-delivered with DC vaccines or integrated into delivery vectors.

Checkpoint blockade helps restore DC functionality within tumors. Programmed death-ligand 1 (PD-L1) is often upregulated on tumor-infiltrating DCs and suppresses T cell activation. Blocking PD-1/PD-L1 interactions allows DCs to engage with naive and memory T cells more effectively [40]. Additionally, DC trafficking remains a limitation, particularly for moDCs. Efforts to increase CCR7 expression or precondition injection sites with chemokines such as CCL19 have improved lymph node migration and enhanced vaccine responses.

Optimizing DC design to resist immunosuppression, respond to inflammatory stimuli, and reach appropriate anatomical sites will be essential for the success of DC-based vaccines in the clinical setting.

### Limitations of DC Vaccines

**Fig. 3:**
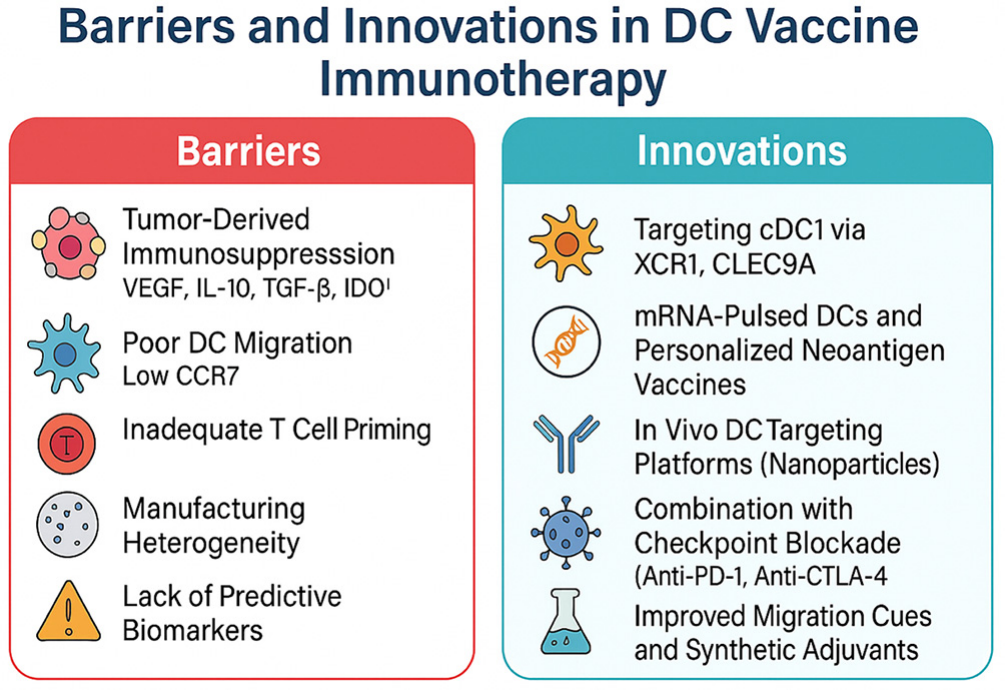
Barriers and innovations in dendritic cell vaccine immunotherapy. This figure contrasts the immunosuppressive barriers that limit dendritic cell vaccine efficacy with emerging strategies designed to overcome them. Barriers include tumorderived cytokines (*e.g.*, IL-10, VEGF), reduced DC migration, and T cell dysfunction. Innovative solutions involve cDC1 targeting, mRNA-based antigen delivery, adjuvant integration, and combination therapies with immune checkpoint inhibitors. These approaches aim to enhance T cell priming and durable antitumor responses.

DC-based vaccines have been extensively studied and clinically tested across a wide range of malignancies. However, their translation into consistently effective cancer therapies remains limited due to multiple biological, logistical, and clinical challenges (Fig. 3). Understanding these limitations is essential for guiding the next generation of vaccine development. Although DC vaccines elicit immune responses in many patients, they rarely induce objective tumor regressions when used alone. In cancers such as glioblastoma, prostate cancer, or pancreatic cancer, DC monotherapy often fails to generate robust effector responses capable of immunosuppression. Response rates vary widely and frequently depend on tumor type, antigen load, and immune contexture [41, 42].

The TME actively disrupts DC function. Tumor cells and stromal components secrete immunosuppressive factors such as VEGF, IL-10, TGF-β, PGE2, and IDO, which inhibit DC maturation and promote tolerogenic phenotypes. This immunosuppressive milieu can prevent DCs from upregulating costimulatory molecules and presenting antigen efficiently. Additionally, tumor-infiltrating DCs often express PD-L1 or fail to produce IL-12p70, which skews T cell responses toward exhaustion or tolerance.

The ability of *ex vivo*-generated DCs to reach secondary lymphoid organs is often suboptimal. DCs generated from monocytes under standard GMP protocols express low or variable levels of CCR7, which is necessary for homing to lymph nodes via the CCL19/CCL21 axis. As a result, many administered DCs fail to engage effectively with naive T cells, reducing vaccine potency [43].

Monocyte-derived DCs display heterogeneity in MHC class I and II presentation, depending on donor variability, cytokine exposure, and the maturation stimulus used. Inconsistent expression of antigen-processing machinery, such as TAP and proteasomal components, can lead to suboptimal presentation of tumor epitopes. Moreover, DCs pulsed with long peptides or whole lysates may fail to process antigens into immunodominant epitopes unless appropriate intracellular routing is achieved [44]. Clinical trials have lacked standardized assays to evaluate DC functionality, antigen presentation, and T cell priming capacity. Current immune monitoring tools are insufficient to predict which patients will benefit from DC vaccines. This gap complicates trial design, regulatory review, and patient stratification. Without validated potency markers, manufacturing and release criteria remain heterogeneous. The production of autologous DC vaccines requires leukapheresis, cell culture, antigen loading, maturation, quality control, and cryopreservation [45]. Each of these steps introduces technical variability and demands GMP-level infrastructure. Batch-to-batch variability, resource constraints, and limited scalability hinder broad clinical implementation and increase costs relative to off-the-shelf biologics [46]. DC vaccines that present a narrow set of tumor antigens may fail to capture the full heterogeneity of neoantigen expression within the tumor. Immune escape through antigen loss variants, HLA downregulation, or defective antigen processing can diminish vaccine efficacy. Broader epitope coverage and the use of personalized neoantigen pools are critical in addressing tumor evolution and immune editing [47]. To overcome these challenges, researchers are exploring *in vivo* targeting of naturally circulating DC subsets, improved antigen selection algorithms, novel adjuvant formulations, and integration with checkpoint blockade or metabolic modulators. Continued efforts to streamline manufacturing, identify predictive biomarkers, and optimize functional assays will be essential for maximizing the therapeutic potential of DC vaccines in oncology.

### Clinical Trials and Translational Advances

Over the past two decades, DC vaccines have been tested in various clinical trials across a broad range of malignancies, including prostate cancer, melanoma, glioblastoma, and non-small cell lung cancer (Table 2). These studies have demonstrated consistent safety profiles and immunogenicity, although objective response rates have varied [48].

The approval of sipuleucel-T for metastatic prostate cancer represented the first regulatory milestone for a DC-based immunotherapy [49]. This vaccine used autologous PBMCs, including DCs, pulsed with a fusion protein of prostatic acid phosphatase and GM-CSF. Although the overall survival benefit was modest, sipuleucel-T validated the principle of DC vaccination.

Melanoma remains the most thoroughly studied indication. Multiple trials have shown that autologous moDCs pulsed with melanoma antigens can generate durable T cell responses, particularly when combined with checkpoint blockade [50]. Neoantigen-based DC vaccines have shown promising results in phase I trials, where they induced polyfunctional T cells and clonal expansion [31].

In glioblastoma, DCVax-L, a lysate-pulsed autologous DC vaccine, demonstrated long-term survival in a subset of patients. An interim analysis of a large phase III trial indicated improved median survival in patients with minimal residual disease [51]. These results have increased interest in integrating DC vaccines into standard treatment regimens.

Emerging technologies have improved vaccine design. Single-cell RNA sequencing allows profiling of DC subsets and helps identify exhaustion markers or suppressive gene signatures [52]. Bioinformatics tools now guide neoantigen selection based on predicted MHC binding, epitope stability, and T cell receptor affinity. Machine learning approaches have further refined immunogenicity prediction [53]. Translational bottlenecks include labor-intensive manufacturing, batch variability, and limited access to GMP-compliant facilities. To address these issues, researchers are exploring decentralized vaccine production hubs and synthetic DC mimetics using biomaterials. These systems offer scalable, off-the-shelf solutions that preserve DC-like function while avoiding the complexity of live cell processing.

Future directions involve tailoring DC vaccines to immune phenotype, using personalized combination therapies, and employing integrated biomarkers to predict response. DC vaccines are expected to complement T cell-based therapies and serve as adjuvants in multipronged immuno-oncology strategies.

## Conclusion

DC-based vaccines represent a promising platform for cancer immunotherapy by bridging innate and adaptive immune responses. Advances in subset characterization, antigen loading strategies, and manufacturing protocols have expanded the potential of DC vaccines in both personalized and off-the-shelf formats. Despite demonstrated safety and immunogenicity in clinical trials, their therapeutic efficacy remains limited by factors such as poor lymph node migration, tumor-induced immunosuppression, and antigen presentation inefficiencies.

Recent innovations aim to address these barriers through the use of specialized DC subsets, such as cDC1s, integration with immune checkpoint inhibitors, and nanoparticle-based delivery systems. Personalized neoantigen targeting and *in vivo* DC programming have further improved the specificity and potency of DC-mediated immune responses. Continued refinement of vaccine formulations, identification of predictive biomarkers, and combinatorial approaches are expected to enhance clinical success and broaden the application of DC-based immunotherapies.

As our understanding of DC biology deepens, future vaccine strategies will likely incorporate modular, scalable, and patient-specific components to improve therapeutic outcomes across cancer types. Dendritic cells remain a cornerstone of translational immunology and a vital component of next-generation cancer immunotherapies.

## Figures and Tables

**Table 1:** Functional summary of dendritic cell subsets.

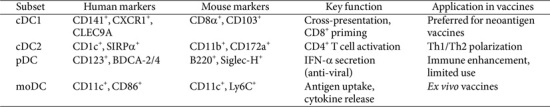

**Table 2:** Clinical trials of DC vaccines in cancer (selected examples).